# Thoughts From the Trenches: Should We Look at the “Healthy”?

**DOI:** 10.3389/fpubh.2020.00490

**Published:** 2020-09-09

**Authors:** Víctor M. Martínez-Taboada, Marcos López-Hoyos, Javier Crespo, Pedro Muñoz Cacho, José L. Hernández

**Affiliations:** ^1^Division of Rheumatology, Hospital Marqués de Valdecilla-IDIVAL, Santander, Spain; ^2^University of Cantabria, Santander, Spain; ^3^Division of Immunology, Hospital Marqués de Valdecilla-IDIVAL, Santander, Spain; ^4^Division of Gastroenterology, Hospital Marqués de Valdecilla-IDIVAL, Santander, Spain; ^5^Department of Medicine and Psychiatry, Gerencia de Atención Primaria, Servicio Cántabro de Salud, Santander, Spain; ^6^Department of Internal Medicine, Hospital Marqués de Valdecilla-IDIVAL, Santander, Spain

**Keywords:** SARS-CoV-2 (virus), Spain, serological tests, research, COVID-19

## Introduction

The pandemic triggered after the massive spread of SARS-CoV-2 from Wuhan (1) is hitting most countries with varying degrees of virulence, striking particularly hard in Spain. A pandemic without precedent, and of a magnitude and severity unknown to this century. Let's contrast and compare some recent data on other seasonal respiratory viruses with serious health consequences. During the 2018–2019 influenza epidemic in Spain, there were 490,000 non-serious cases, 35,300 hospitalizations, 2,500 patients were admitted to the intensive care units (ICU) and 6,300 died (https://vacunasaep.org). Concerning SARS-CoV-2 infection, on May 15, 2020, the Spanish Ministry of Health (https://www.mscbs.gob.es) had reported 230,183 cases, 124,571 hospitalizations, 11,493 ICU admissions, and 27,459 deaths. Therefore, the numbers speak for themselves when both infections were compared.

It is true that the Spanish Healthcare System has some peculiarities that may have made initial control of the pandemic difficult. These include the system fragmentation into 17 health regions and the absence of a proactive strategy to tracing contacts or search for potential cases, coupled with an absence of preventive measures to foresee the supply chain shortages for personal protection equipment and diagnostics tests (RNA extraction reagents and RT-PCR kits). Nevertheless, the Spanish Healthcare System has demonstrated great flexibility and adaptability during pandemics. In fact, hospital beds and intensive care facilities have increased, even external hospitalization centers such as the IFEMA in Madrid with up to 5,000 beds (an ~39% increase in the number of hospital beds in one of the most affected regions in Spain) have been set up in record time to attend COVID-19 patients, and a number of research laboratories (24 laboratories accreditated by the National Research Institute Carlos III) got ready to do RT-PCR diagnostics test across the country. The Spanish civil society has also demonstrated a great responsibility in adopting all the preventive measures taken by the health authorities. Besides, our knowledge of the best therapeutic measures to fight against coronavirus is rapidly increasing. Therefore, there is likely to be room for hope even in the case of a second SARS-CoV-2 wave in our country.

Without a doubt, the economic impact in a system highly dependent on the tourism and service sectors will be enormous, with the consequent impoverishment and added difficulty in taking public health actions. At the time of writing this manuscript, and with the confinement period just finished, the Spanish government, advised by experts, has implemented a number of measures intended to gradually start the normal daily activities. These actions are accompanied by increased SARS-CoV-2 testing to know the degree of immunization in the general population (2). However, within the first weeks of “*normal activity*” we are seeing an ever increasing number of traceable outbreaks and, in some cases, they are at risk of developing as community epidemics in a second wave.

## Will the Sanitary Passport Clear all our Doubts?

Due to several reasons, we deem that classifying the individuals into immunized and non-immunized groups will not solve all our troubles as most people believe:

(a) At present, not only do we not know the real prevalence of the viral infection, but more importantly, we do not know what the progression of the pandemic will be. We also do not know whether subjects who have had COVID19 will be immune in a possible future SARS-CoV-2 wave, probably next winter (3). Besides, we still do not appreciate the mutation capacity of SARS-CoV-2 and its impact on infectivity and lethality. Preliminary data from Spain big survey on seroprevalence indicates 5.2% overall humoral immunity (4).

(b) There are some doubts about the sensitivity and specificity of SARS-CoV-2 serological tests, as well as about the SARS-CoV-2 epitopes they include. Thus, these tests should be interpreted with caution, especially in the case of lateral flow rapid tests (5).

(c) Although the original intention is on massive population-wide testing, the impossibility of carrying out this measure in the entire Spanish population is clear. From the outset, it seems reasonable to select the high-risk groups for testing: elderly people (especially nursing homes residents), immunosuppressed patients, health care workers, etc. (6). A different issue arises when a low-risk population is considered: Are we going to force the entire population to test? Are we going to test all the children or youth people? In fact, although there is little information (7) they are probably the main asymptomatic groups and potentially spreaders of the disease to high-risk individuals. Besides, some experts consider that summer is a season with a lower incidence of viral infections not only because of climatic factors but also because, with schools closed, viruses have much less ability to spread, as it has happened with other viral outbreaks.

(d) Therefore, not only are there factors dependent on the virus, but we also ought to take into account host factors. Not everyone may be capable of developing an effective immune response against the infection. It is also unclear what level of antibodies one should reach, or whether this level could protect against a second encounter with the virus or any of its variants (8). If the outbreak becomes endemic-seasonal, the possibility of response might also decrease over time. On the other hand, recent evidences point to the protective role of cellular immunity, which has not been taken into account until very recently, either cross-reactive with seasonal coronavirus (9) or after SARS-CoV-2 infection (10).

(e) Something very similar would happen if we hopefully had an effective vaccine. It might not protect everyone, or at least not equally. Furthermore, the duration of a proper immunization is also unknown. Therefore, until we increase our knowledge about immunity generated by SARS-CoV2, continuing to work on general preventive actions and antiviral treatments seems to be the key to success against this infection. The use of facial mask and hand washing, together with social distancing, will remain the main actions in counteracting the pandemic.

## Discussion

Given the current uncertainty derived from the lack of knowledge of the natural history of the viral disease, we can anticipate two extreme scenarios:

1. Favorable scenario: a virus with little ability to mutate, the development of permanent humoral and cellular immunity, adequate preventive actions (including general measures, effective pharmacological interventions, and vaccines), the discovery of effective antiviral drugs, and no appearance of another breakthrough pandemic.
2. Unfavorable scenario: a virus with high capacity for mutation, the development of transient immunity, inadequate preventive measures, the lack of effective antiviral agents, or a new pandemic breaking out.

It is crucial to bear in mind that it is extremely complicated for all the favorable conditions to eradicate COVID19. Nonetheless, the presence of a single unfavorable condition can be critical in the general outcome of the pandemic.

So far in Spain, only those patients with moderate-severe symptoms have been tested for coronavirus with PCR tests. The health system has made a heroic effort to treat those patients with serious COVID19, and several drugs have been used with a rational pathophysiological principle, despite the lack of solid scientific evidence to date (11). On the other hand, not only in Spain but in the rest of the world, numerous well-designed studies have been launched in a short period of time, to demonstrate whether the different therapeutic agents we are currently using can really be effective in treating COVID19. It stands to reason that knowing the real efficacy of these therapeutic agents will help patients who become infected in the future to overcome their disease and design treatment protocols according to its severity, although today none of these agents seems to have a curative role. Only the collective effort of the international scientific community, through the careful study of SARS-CoV-2 characteristics, the intimate pathogenic mechanisms of the disease, the pattern of the different responses of the host to infection, and the protective health measures against it, would help to control this pandemic promptly.

The possibilities that we are going to face and the different clinical scenarios in which we have to fight to overcome COVID19 are shown in the Table 1. The war against this serious disease, as previously discussed, is still being waged without truce. This fight will provide relevant information for new patients with severe COVID19 manifestations: identifying prognostic factors for disease severity and progression to ARDS (12, 13), and determine the real efficacy of therapeutic options through well-designed clinical trials (14). The study of mild cases of the disease and the asymptomatic carriers might help us answer other relevant questions, such as the duration of the contagious period, the characteristics of the non-severe disease or even the type, intensity and duration of immunization (8). The issue of immunization is relevant in the development of vaccination strategies once they are ready to be used.

**Table 1:** Possible scenarios, groups susceptible to intervention, and opportunities in SARS-CoV-2 infection.

**Individuals**	**Scenario**		**Susceptible to intervention**	**Opportunities**
INFECTED	- Clinical*+/Test +	- Serious disease. Hospital admission - Mild disease. Outpatient treatment	No	- Study available and new treatment options (randomized controlled trials)- Identify prognostic factors for disease severity and progression to ARDS- Determine the characteristics for non-severe disease- Determine contagious period- Determine duration of immunization
	- Clinical +/Test -	- False negative test result - COVID19 ruled out (others URTI)	Yes	- Improve molecular diagnostic tests (conventional RT-PCR, Xpert RT-PCR, LAMP)- Identify (**) and prophylaxis (?)
	- Clinical +/Test ND	- Mild disease. Outpatient control - COVID19 ruled out (others URTI)	Yes	- Identify and determine the characteristics for non-severe disease - Identify and prophylaxis (?)
	- Clinical—/ Test ND	- Asymptomatic carriers	No	- Identify and determine characteristics for non-severe disease- Determine the duration of immunization
“SUPPOSEDLY NOT INFECTED”	- Clinical—/Test ND	- COVID19 contact +/No high-risk group	Yes	- Identify and determine immune status- Prophylaxis accordingly
	- Clinical—/Test ND	- COVID19 contact+/High-risk group *(elderly, immunosuppressed patients, heath care workers, etc.)*	Yes	- Identify and determine immune status- IgM—/ IgG—(mandatory prophylaxis)
	- Clinical—/Test ND	- No contact/No high risk group *They are impossible to distinguish from asymptomatic carriers*	Yes (?)	- Identify and determine immune status (?)-Prophylaxis accordingly (?)
	- Clinical—/Test ND	- No contact/High-risk group *(elderly, immunosuppressed patients, health care workers, etc.)*	Yes	- Identify and determine immune status- IgM—/IgG—(mandatory prophylaxis)

*Clinical, clinical manifestations suggestive of respiratory tract infection of any cause; Test, means RT-PCR; ND, not done; URTI, upper respiratory tract infection; ARDS, acute respiratory distress syndrome; RT-PCR, real time quantitative polymerase chain reaction; LAMP, loop-mediated isothermal amplification). (**) Identify, means to determine immunization status through reliable serological tests.

A critical part of the fight against SARS-CoV-2 is the improvement in diagnostic tests, not only during the acute phase of the disease, but also in the sensitivity and specificity of serological tests (6, 15). Furthermore, the development of more suitable tests to determine the cellular immunity against the virus, will improve the knowledge of the disease and the opportunities for intervention. This is a key aspect, especially in high risk groups, where in the absence of curative treatments for the infection, establishing appropriate prophylaxis measures is crucial. Nowadays, and pending the results of appropriate studies (antimalarial drugs, vitamin D, trained immunity, etc.) (16–19), the only preventive measures that have been proven effective are the implementation of precautions and hygienic measures to minimize human transmission of the virus, especially in high-risk populations.

The investigation focused on “healthy” people who have contacted the virus and did not develop the disease, or those who have been cured without problems (including the asymptomatic carriers) is a crucial issue (9). Probably, this may be the key to identifying new therapeutic or preventive interventions. Perhaps, we ought to go for strategies that combine the study of the “sick” and the “healthy” individual. In the meantime, it is probably utopian to reach the entire population with an appropriate diagnostic test, but we must prepare ourselves to reach the maximum number of people, with a logistical and economic effort of unprecedented dimensions. At this moment, probably the most cost-effective approach will rely in the urgent identification of cases and outbreaks by RT-PCR together with searching of contacts to trace any focus (20). However, this will all be investment and not spending. As it is evident, we have a lot of work to do but, at the same time, a lot of opportunities to explore.

## Author Contributions

VM-T designed the manuscript. VM-T, JH, ML-H, JC, and PC wrote the manuscript. All authors discussed the results and contributed to the final paper.

## Conflict of Interest

The authors declare that the research was conducted in the absence of any commercial or financial relationships that could be construed as a potential conflict of interest.
